# Outcomes of induction versus spontaneous onset of labour at 40 and 41 GW: findings from a prospective database, Sri Lanka

**DOI:** 10.1186/s12884-022-04800-1

**Published:** 2022-06-27

**Authors:** Hemantha Senanayake, Ilaria Mariani, Emanuelle Pessa Valente, Monica Piccoli, Benedetta Armocida, Caterina Businelli, Mohamed Rishard, Benedetta Covi, Marzia Lazzerini

**Affiliations:** 1 University Obstetrics Unit, De Soysa Hospital for Women, Colombo, Sri Lanka; 2 Faculty of Medicine, Department of Obstetrics & Gynaecology, University of Colombo, Colombo, Sri Lanka; 3 Institute for Maternal and Child Health - IRCCS “Burlo Garofolo”, WHO Collaborating Centre for Maternal and Child Health, Via dell’Istria 65/1, 34137 Trieste, Italy; 4 Department of Obstetrics & Gynaecology, Institute for Maternal and Child Health - IRCCS “Burlo Garofolo”, Trieste, Italy; 5 Department of Cardiovascular, Endocrine-metabolic Diseases and Aging, National Institute of Health, Rome, Italy

**Keywords:** Induction of labour, Full term pregnancy, Late term pregnancy, Pregnancy outcomes, Low risk pregnancies

## Abstract

**Objectives:**

The World Health Organization recommends induction of labour (IOL) for low risk pregnancy from 41 + 0 gestational weeks (GW). Nevertheless, in Sri Lanka IOL at 40 GW is a common practice. This study compares maternal/newborn outcomes after IOL at 40 GW (IOL40) or 41 GW (IOL41) versus spontaneous onset of labour (SOL).

**Methods:**

Data were extracted from the routine prospective individual patient database of the Soysa Teaching Hospital for Women, Colombo. IOL and SOL groups were compared using logistic regression.

**Results:**

Of 13,670 deliveries, 2359 (17.4%) were singleton and low risk at 40 or 41 GW. Of these, 456 (19.3%) women underwent IOL40, 318 (13.5%) IOL41, and 1585 (67.2%) SOL. Both IOL40 and IOL41 were associated with an increased risk of any maternal/newborn negative outcomes (OR = 2.21, 95%CI = 1.75–2.77, *p* < 0.001 and OR = 1.91, 95%CI = 1.47–2.48, *p* < 0.001 respectively), maternal complications (OR = 2.18, 95%CI = 1.71–2.77, *p* < 0.001 and OR = 2.34, 95%CI = 1.78–3.07, *p* < 0.001 respectively) and caesarean section (OR = 2.75, 95%CI = 2.07–3.65, *p* < 0.001 and OR = 3.01, 95%CI = 2.21–4.12, *p* < 0.001 respectively). Results did not change in secondary and sensitivity analyses.

**Conclusions:**

Both IOL groups were associated with higher risk of negative outcomes compared to SOL. Findings, potentially explained by selection bias, local IOL protocols and CS practices, are valuable for Sri Lanka, particularly given contradictory findings from other settings.

**Supplementary Information:**

The online version contains supplementary material available at 10.1186/s12884-022-04800-1.

## Introduction

Over the past decades, induction of labour (IOL) rates have continued to rise, with a reported average incidence of one out of four births at term (from 37 + 0 gestational weeks [GW]) in high-income countries, and very similar rates in low and middle-income countries (LMIC) [1]. According to the World Health Organization (WHO), IOL should be performed only when there is a clear medical indication and the expected benefits outweigh its potential harms [2]. As perinatal risks increase with gestational age, the current recommendation from WHO, the National Institute for health and Care Excellence (NICE), and most scientific societies is to perform IOL in women who are known with certainty to have reached 41 GW (i.e., from 41 + 0 GW) [1, 3–6].

However, especially in the last few years, the debate on optimal timing for IOL and, specifically, whether IOL around term improves birth outcomes, has become very lively. The most recent Cochrane review (2018) including 30 randomized clinical trials (RCTs), seven conducted in southeast Asia, highlighted that IOL from 37 GW compared to expectant management is associated with fewer perinatal deaths, neonatal intensive care unit admissions, babies with low Apgar score and caesarean sections (CS), but also with more operative vaginal deliveries (OVD) [7]. Authors concluded that further investigation is needed into optimal timing of IOL, together with exploration of women’s risk profiles and preferences [7].

More recently, other evidence has emerged. In 2019, a meta-analysis of cohort studies including 15 million pregnancies in high-income countries reported that stillbirth increases slightly but significantly from 37 GW onward with a 64% increase in the risk of stillbirth at 41 GW compared to 40 GW [8], thus suggesting the opportunity of elective IOL even before the traditional cut-off of 41 GW.

Other relevant RCTs were published in parallel. A single-centre RCT in the UK among nulliparous women over 35 years old without complications showed no significant difference in maternal and newborn outcomes between IOL at 39 GW and expectant management [9]. More recently, the ARRIVE trial, a multicentre RCT conducted by Grobman et al. among 6106 low-risk nulliparous women in the US compared IOL at 39 GW to expectant management and found lower incidence of CS with IOL (RR 0.84; 95%CI 0.76–0.93) and no significant differences in perinatal deaths or severe neonatal complications (RR 0.80; 95%CI 0.64–1.00) [10]. A meta-analysis of cohort studies [11] confirmed the results of this trial [10].

Two other RCTs in uncomplicated singleton pregnancies—INDEX, a Dutch trial enrolling 1801 women [12], and SWEPIS, a Swedish multicentre trial in 14 hospitals including 2760 women [13]—found that IOL at 41 GW was associated with fewer adverse perinatal outcomes than expectant management until 42 GW [12, 13]. Notably, the SWEPIS study was stopped early because of higher perinatal mortality with SOL [13].

On the other hand, a national retrospective register-based cohort study evaluating the effects of changes in routine elective IOL policies in Denmark (42 GW versus 41 + 3 and 41 + 5 GW) found no differences in neonatal outcomes including stillbirth, despite the number of women with IOL increasing significantly [14]. Additionally, a systematic review reported that IOL at 41 versus 42 GW was associated with an increased risk of CS (RR 1.11; 95%CI 1.09–1.14) and adverse maternal outcomes [15].

In conclusion, evidence is still contradictory and the debate is quite polarized. No clear context-specific evidence exists on women’s preferences on IOL. The ARRIVE trial reported that US women in the IOL group had a positive perception of increased control over birth [10, 16], while other qualitative systematic reviews concluded that the majority of women feared medical interventions, preferring a physiological birth promoting their physical and psychosocial capacities [16, 17].

In addition, literature on outcomes of IOL around term versus expectant management in LMIC is very scarce. According to the WHO Global Survey on Maternal and Perinatal Health, IOL was performed in Asia in 12.1% of deliveries and associated with negative neonatal outcomes [18]. According to existing estimates, Sri Lanka has the highest IOL rate in Asia (about 35.5% of total deliveries) [1, 18] with 77.2% of all IOL being elective [18].

Elective IOL at 40 GW is often clinically justified by local professionals on the basis of supposed earlier loss of foeto-placental function in South Asian populations compared with Caucasian women or Asian counterparts, and on the fear of increased risk of foetal morbidity [19–21]. Nevertheless, no study from Sri Lanka has so far explored outcomes of women or newborns with IOL at 40 GW versus 41 GW.

The main objective of this study was to compare the absence of a maternal or neonatal complications between low-risk women induced at 40 GW and those in spontaneous onset of labour (SOL) at 40 or 41 GW. Secondary objectives were to compare the absence of maternal or neonatal complications between women induced at 41 GW and those in SOL at 40 or 41 GW; and to compare the mode of delivery between induced women and those in SOL. Data for this study were collected over four years in a prospective individual patient database established in 2015 at the De Soysa Teaching Hospital for Women, Colombo, the largest maternity hospital in Sri Lanka.

## Methods

### Study design

This is an observational study reported according to the STrengthening the Reporting of OBservational studies in Epidemiology (STROBE) statement (Additional Table 1) [22].

**Table 1:** Characteristics of the study population

Population	IOL at 40 GW (40 + 0 to 40 + 6) *N* = 456 n (%)	IOL at 41 GW (41 + 0 to 41 + 6) *N* = 318 n (%)	SOL (40 + 0 to 41 + 6) *N* = 1585 n (%)
Maternal Age			
< 35 years	401 (87.9)	290 (91.2)	1424 (89.8)
≥ 35 years	55 (12.1)	28 (8.8)	161 (10.2)
Education			
None	1 (0.2)	2 (0.6)	2 (0.1)
Primary	10 (2.2)	4 (1.3)	26 (1.6)
Secondary	353 (77.4) ^a^	266 (83.6)	1341 (84.6)
Higher	91 (20.0) ^a^	46 (14.5)	211 (13.3)
Missing	1 (0.2)	0	5 (0.4)
Working status			
Working	81 (17.8)	48 (15.1)	227 (14.3)
Housewife	370 (81.1)	270 (84.9)	1344 (84.8)
Missing	5 (1.1)	0	14 (0.9)
Marital status			
Married	451 (98.9)	311 (97.8)	1570 (98.6)
Unmarried	3 (0.7)	7 (2.2) ^b^	14 (0.9)
Unmarried living together	1 (0.2)	0	2 (0.1)
Missing	1 (0.2)	0	6 (0.4)
Parity			
0	260 (57.0) ^a^	198 (62.3) ^b^	754 (47.6)
≥ 1	196 (43.0) ^a^	120 (37.7) ^b^	831 (52.4)
Asian criteria-based BMI [25]			
Underweight (< 18.4)	38 (8.3)	33 (10.4)	159 (10.0)
Normal (18.5–22.9)	312 (68.4)	190 (59.7) ^b^	1061 (67.0)
Overweight (23–27.4)	106 (23.2)	95 (29.9) ^b^	365 (23.0)
Operator delivering care			
Nurse	200 (43.9) ^a^	116 (36.5) ^b^	899 (56.7)
Midwife	110 (24.1)	101 (31.8)	431 (27.2)
House Officer	4 (0.9)	4 (1.3)	24 (1.5)
Mid-level staff ^c^	140 (30.7) ^a^	96 (30.2) ^b^	224 (14.1)
Consultant	2 (0.4)	1 (0.3)	3 (0.2)
Missing	0	0	4 (0.3)
Neonatal weight at birth			
2000	0	0	0
2000 to 2499	13 (2.9)	3 (0.9) ^b^	55 (3.5)
2500 to 3499	374 (82.0)	246 (77.4)	1278 (80.6)
3500 to 4000	57 (12.5)	61 (19.2) ^b^	234 (14.8)
> 4000	11 (2.4) ^a^	8 (2.5) ^b^	13 (0.8)
Missing	1 (0.2)	0	5 (0.3)

*Abbreviations*: *BMI* Body mass index, *GW* Gestational weeks. *IOL* Induction of labour, *SOL* Spontaneous onset of labour

^a^Statistically significant *p* value (*p* < 0.05) in the comparison IOL at 40 GW vs SOL; ^b^ Statistically significant *p* value (*p* < 0.05) in the comparison IOL at 41 GW vs SOL; ^c^ Mid-level staff defined as Senior House Officer or Registrar

### Population and setting

Data collection, data quality assurance procedures and standard operating procedures used for the individual patient database are reported elsewhere [23]. Briefly, 150 variables (i.e., maternal sociodemographic characteristics, risk factors, process indicators, maternal and neonatal outcomes) were collected for each birth on two wards of the University Obstetric Unit at De Soysa Teaching Hospital for Women, using a standardised two-page form, and entered in real time in an electronic database. De Soysa is the largest referral hospital for maternity care in Sri Lanka and all deliveries occurring in these two wards from May 2015 to May 2019 were entered in the database and considered for inclusion. Overall data quality was routinely monitored with external independent random review of 5% of forms and 5% of entered births to maintain an error rate in data collection below 0.02% [24]. Data were also externally monitored for completeness and internal consistency at roughly 4-month intervals [23].

We included “low risk women” with singleton pregnancies and a foetus in cephalic presentation whose delivery occurred between 40 + 0 and 41 + 6 GW. We excluded all cases with any maternal or foetal characteristics which may have affected outcomes, such as: maternal obesity (Asian criteria-based body mass index -BMI- more than 27.5 [24]), previous CS, macrosomia at ultrasonography (defined as estimated birthweight exceeding the 90^th^ centile for gestational age), hypertension disorders during pregnancy (i.e., pregestational or gestational hypertension, preeclampsia, eclampsia, HELLP syndrome), chorioamnionitis, major foetal malformations, intrauterine growth restriction at ultrasonography (IUGR), small for gestational age (SGA), pre-gestational diabetes, gestational diabetes with the need of drug therapy, maternal cardiac disease, maternal hypothyroidism, polyhydramnios, oligohydramnios, antepartum haemorrhage (APH), major placenta praevia, placental accretism, severe anaemia (haemoglobin < 7.0 g/dl) and other foetal and maternal pathological conditions, i.e., systemic lupus erythematosus, pre-pregnancy deep venous thrombosis, epilepsy, suspected cephalo pelvic disproportion, recurrent infection, pancreatitis or glomerulonephritis in pregnancy, chickenpox disease, chronic disease, signs of potentially impaired foetal wellbeing (non-reassuring or pathological cardiotocography, reduced foetal movement, meconium stained amniotic fluid). We also excluded macerated stillbirth before 41 + 0 GW, as those births are routinely induced. All women with a reported indication for IOL suggesting the presence of maternal or foetal characteristics described above, such as diabetes, macrosomia at ultrasound, IUGR/SGA, were excluded from the analysis.

### Comparison groups and outcomes

We compared women with IOL at 40 GW (40 + 0 to 40 + 6 GW), women with IOL at 41 GW (41 + 0 to 41 + 6 GW), and women with SOL in between 40 + 0 to 41 + 6 GW. Artificial separation of membranes alone was not considered as induction. Low risk women with prelabour rupture of membranes were included in the SOL group.

The main outcome is the absence of “negative outcomes”, defined in line with previous literature [2, 3, 7] as any birth that included an intervention (i.e., CS, OVD) and/or a maternal or neonatal complication (i.e., not completely physiological).

As listed in Additional Table 2, maternal complications included in the definition of negative outcomes were: abruptio placentae, amniotic fluid embolisms, cord prolapse, hysterectomy, intensive care unit admission, maternal death, near miss (defined as severe disease such as pre-eclampsia, eclampsia, sepsis, uterine rupture; critical interventions such as Intensive Care Unit admission, intervention radiology, laparotomy, blood transfusion; or organ dysfunction), operative theatre admission after delivery, perineal tears 3rd-4th degree, postpartum haemorrhage (defined as a blood loss above 500 ml), sepsis or severe infection, uterine rupture and other maternal complications not further specified in the database. Included neonatal complications were apgar score less than 5 at 10’, asphyxia (i.e., no spontaneous start of breathing, ventilation for at least 30s and/or thoracic compressions or any drug administration), jaundice with exchange transfusion, major birth trauma (i.e., brachial plexus injury/arm palsy, fractures at any site, sub-aponeurotic hemorrhage), meconium aspiration syndrome, need of feeding support, Neonatal Intensive Care Unit or Special Care Baby Unit admission, neonatal length of stay more than 10 days, perinatal deaths included stillbirth (both macerated and fresh stillbirth based on clinical evaluation), phototherapy for more than 24h (included as a proxy of other neonatal complications such as large for gestational age), respiratory distress syndrome (defined as respiratory distress lasting more than 24h), major neurological complications (e.g., seizures, ventricular hemorrhage), sepsis or infection, ventilation in delivery room and other neonatal complications not further specified in the database.

**Table 2:** Adjusted odds ratios for negative birth outcomes by type of labour

	IOL at 40 GW (40 + 0 to 40 + 6) *N* = 456		IOL at 41 GW (41 + 0 to 41 + 6) *N* = 318		SOL (40 + 0 to 41 + 6) *N* = 1585
	Adj OR (95% CI)	*p* value	Adj OR (95% CI)	*p* value	
Any negative outcome	2.21 (1.75–2.77)	** < 0.001**	1.91 (1.47–2.48)	** < 0.001**	Ref
All maternal complications	2.18 (1.71–2.77)	** < 0.001**	2.34 (1.78–3.07)	** < 0.001**	Ref
Caesarean section	2.75 (2.07–3.65)	** < 0.001**	3.01 (2.21–4.12)	** < 0.001**	Ref
Operative vaginal delivery	1.27 (0.82–1.98)	0.285	0.48 (0.24–0.97)	**0.041**	Ref
Other maternal complications	0.88 (0.55–1.42)	0.606	1.83 (1.19–2.80)	**0.006**	Ref
All neonatal complications	1.63 (1.24–2.14)	** < 0.001**	1.16 (0.83–1.62)	0.370	Ref

*Abbreviations*: *GW* Gestational weeks, *IOL* Induction of labour, *Ref* Reference group, *SOL* Spontaneous onset of labour

ORs are adjusted for age, parity, education, BMI and neonatal weight

Secondary dichotomous outcomes were CS, OVD, maternal complications, neonatal complications.

### Data analysis

Categorical variables were expressed as absolute numbers and compared among groups with χ^2^ or Fisher exact test as appropriate.

We evaluated the association between each group and negative outcome(s), CS, and OVD using multiple logistic regression models adjusting for baseline characteristics i.e., mother age, education, parity [i.e., nulliparous, multiparous], BMI, neonatal weight). Results of logistic regression are also presented for CS and OVD since they were evaluated as clinical outcomes related to failed induction in Sri Lanka [25]. A one-sided Cochran-Armitage test for trend was performed to assess the influence of changes of clinical protocols and staff training practices [26, 27] over different semesters of the study on CS and OVD.

As secondary analyses we compared i) IOL at 40 GW to a group composed of IOL at 41 GW and SOL, in line with analyses by Rydahl and collegues [15], and ii) IOL at 40 GW to IOL at 41 GW. The former analysis allowed the comparison between IOL group at 40 GW and spontaneous labour at the same gestational age, and simultaneously took into account the risks of the ongoing pregnancy including all births at 41 GW, reducing possible bias, while the latter is a comparison of interest in the Sri Lanka setting due to the belief of an earlier loss of foeto-placental function in South Asian populations [19–21].

In addition, since for database construction we were not able to identify if reported hypertensive disorders (pregestational hypertension, preeclampsia, eclampsia, HELLP syndrome), chorioamnionitis, oligohydramnios, APH, and signs of potentially impaired foetal wellbeing (i.e. non-reassuring or pathological cardiotocography, reduced foetal movement, meconium stained amniotic fluid) from 41 + 0 GW were risk factors or complications related to the prolongation of the pregnancy, we performed a sensitivity analysis including women who developed these conditions and considering them as negative birth outcomes.

Data were analysed using STATA version 14.0 (Stata Corporation, College Station TX) and SAS/STAT® software version 9. All statistical tests were two-sided and a p-value less than 0.05 was considered statistically significant.

## Results

### Women’s characteristics

**Fig. 1:**
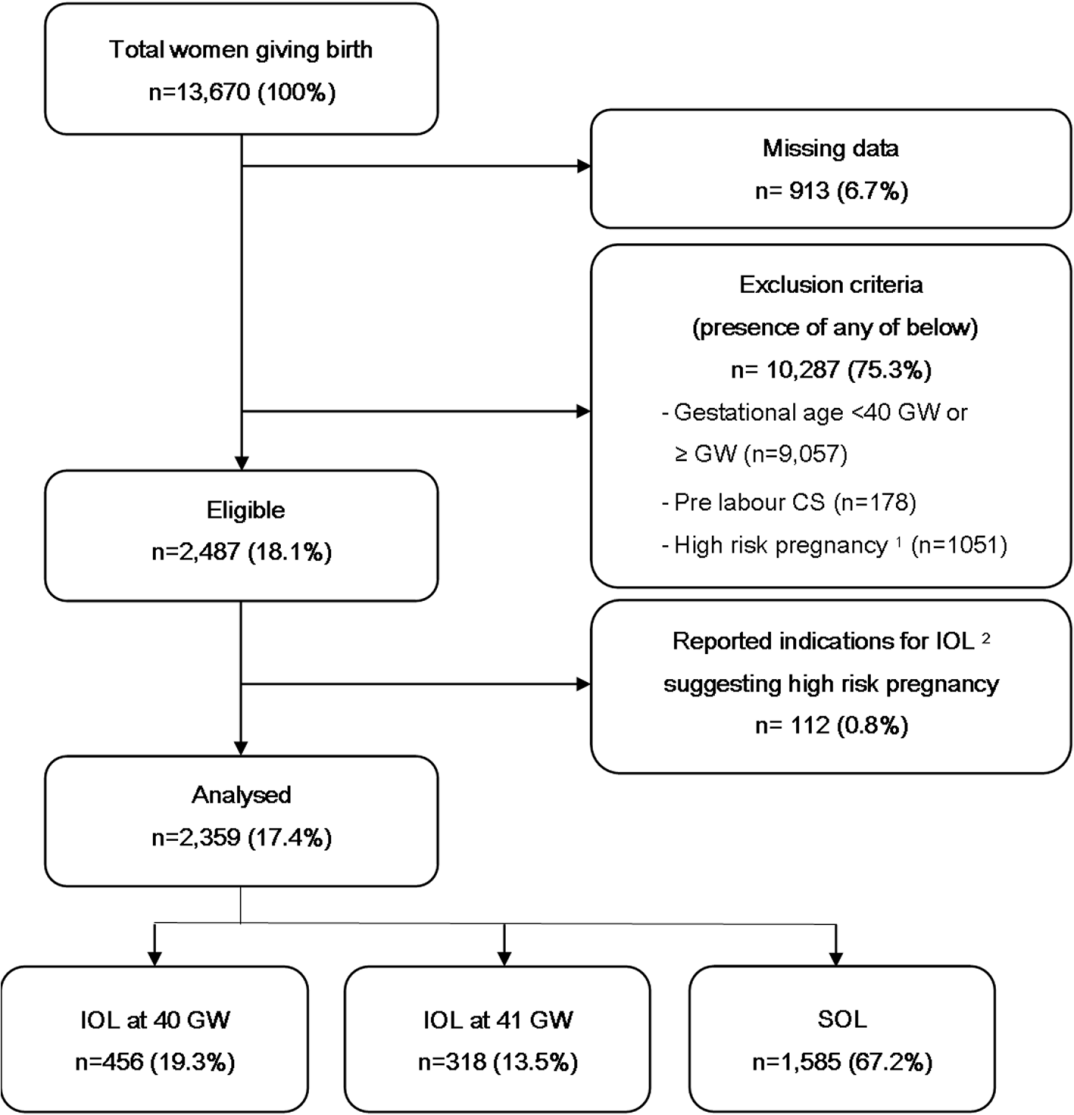
Study sample selection. Notes:^1^ High risk pregnancy defined by the presence of one or more risk factors among: multiple pregnancy, non-cephalic presentations, BMI > 27.5, previous CS, hypertensive disorders (pregestational hypertension, preeclampsia, eclampsia, HELLP syndrome), chorioamnionitis, foetal malformations, IUGR/SGA, pregestational or gestational diabetes in drug therapy, maternal cardiac disease, polyhydramnios, oligohydramnios, APH, severe anaemia, systemic lupus erythematosus, pre-pregnancy deep venous thrombosis, epilepsy, pelvic dysfunction, recurrent infection, pancreatitis or glomerulonephritis in pregnancy, chickenpox disease, chronic disease, signs of potentially impaired foetal wellbeing (i.e. non-reassuring or pathological cardiotocography, reduced foetal movement, meconium stained amniotic fluid). We also excluded macerated stillbirth from the group IOL at 40 GW, as these births are always induced. ^2^ Reported indications for IOL suggesting the presence of the above risk factors. Abbreviations: APH = Antepartum haemorrhage; BMI = Body mass index; CS = Caesarean section; GW = gestational weeks; IOL = induction of labour; IUGR = Intrauterine growth restriction at ultrasonography; SGA = Small for gestational age; SOL = spontaneous onset of labour

A total of 13,670 women delivered in the hospital during the study period. Of these, 2359 (17.4%) matched our inclusion criteria of low-risk singleton pregnancy from 40 + 0 to 41 + 6 GW with the foetus in cephalic presentation (Fig. 1). Among the included women SOL was observed in 1585 women (67.2%), while among 774 cases of IOL, 456 (58.9%) were induced from 40 + 0 to 40 + 6 GW, and 318 (41.1%) from 41 + 0 to 41 + 6 GW. Prostaglandin alone was the most frequent method of induction. It was used for more than 40% of induced women (48.8% in IOL at 40 GW and 43.6% in IOL at 41 GW) followed by artificial rupture of membranes, foley, oxytocin, or a combination of techniques with no differences between groups except for the combination of prostaglandin, oxytocin and artificial rupture of membranes (6.8% in IOL at 40 GW vs 17.5% in IOL at 41 GW, *p* < 0.001) (Additional Table 3).

Some imbalances among groups were observed (Table 1). Women undergoing IOL at 40 GW had a significantly higher level of education compared to the SOL group (20.0% vs 13.3%, *p* = 0.001). Significantly more women were unmarried and overweight in the IOL at 41 GW group compared to SOL (unmarried women: 2.2% vs 0.9%, *p* = 0.040; overweight women: 29.9% vs 23.0%, *p* = 0.009). IOL group at 41 GW had an increased frequency of newborns with a birth weight between 3500 and 4000 g (19.2% vs 12.5% in IOL at 40 GW vs 14.8% in SOL, *p* = 0.035) and above 4000 g (2.5% vs 2.4% in IOL at 40 GW vs 0.8% in SOL, *p* = 0.006). Women with SOL were most often multiparous (52.4% vs 43.0% in IOL at 40 GW vs 37.7% in IOL at 41 GW, *p* < 0.001) and more frequently assisted at delivery by nurses (56.7% vs 43.9% vs 36.5%, *p* < 0.001), while mid-level medical staff (either senior house officers or registrars) was more often involved in IOL groups (30.7% vs 30.2% vs 14.1%, *p* < 0.001).

### Primary outcomes

**Fig. 2:**
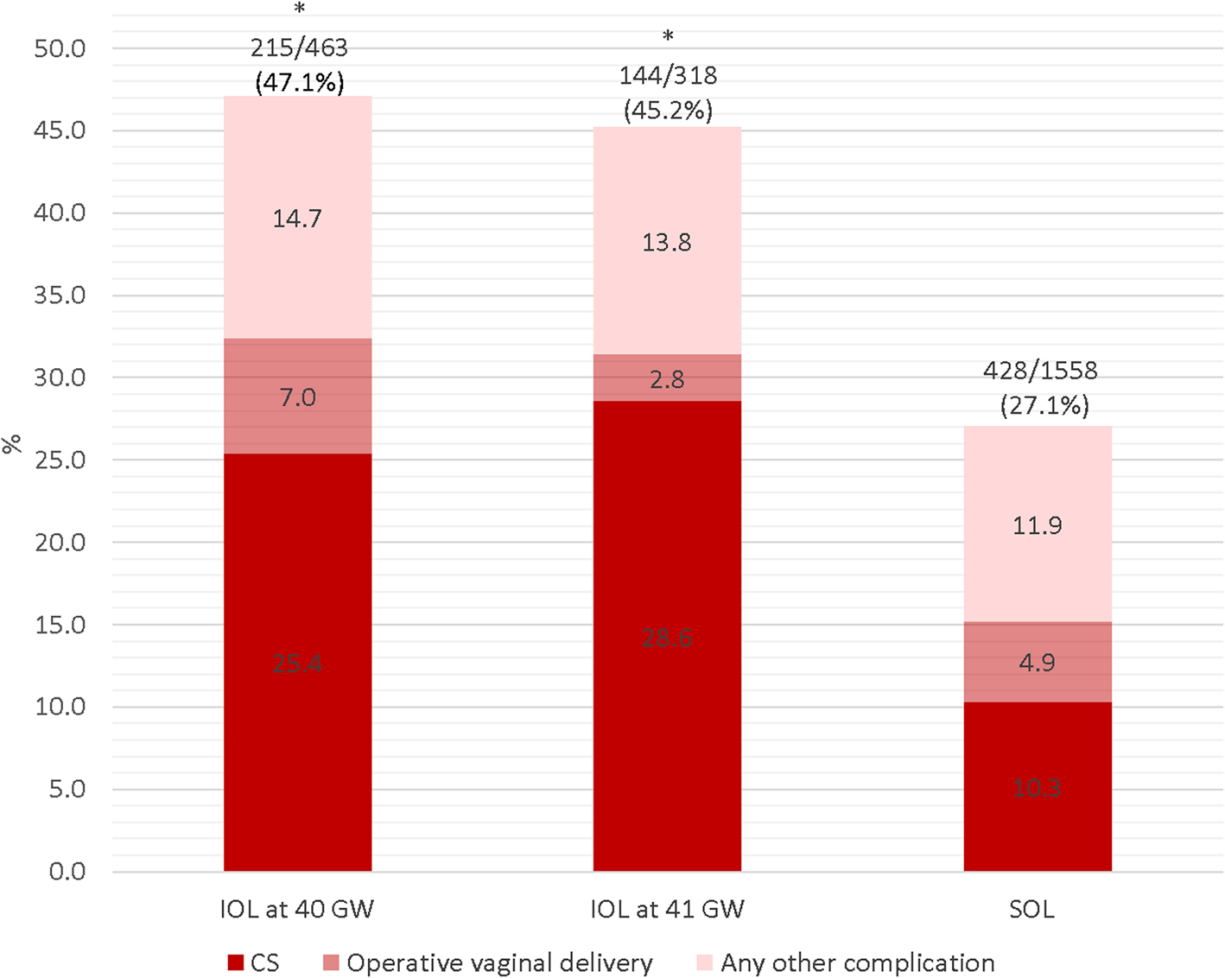
Births with negative outcomes by type of labour. Notes: ^*^ Statistically significant difference (*p* < 0.05) of births with negative outcomes in the comparison with SOL. No significant difference comparing negative outcomes between IOL at 40 to IOL at 41 GW. Abbreviations: CS = Caesarean section; GW = gestational weeks; IOL = Induction of labour; SOL = Spontaneous onset of labour

The overall incidence of births with one or more negative outcomes (including CS and OVD) is reported in Fig. 2. The rate was significantly lower in the SOL group (27.1%, *p* < 0.001) compared to IOL. The CS rate was significantly higher among women undergoing IOL either at 40 GW (25.4%) or at 41 GW (28.6%) when compared with SOL (10.3%, *p* < 0.001). However, no significant differences were found for OVD rate. The proportion of births with any other complication (see Additional Table 2 for the complete list of other complications) was not significantly different among groups (*p* = 0.222). Detailed data is reported in Additional Table 4.

The trend analysis (Additional Fig. 1) showed an increasing CS rate over semesters in the group with IOL at 40 GW only (trend test *p* = 0.021), whereas OVD rate decreased overall (trend test *p* = 0.016) and in IOL at 40 GW (*p* = 0.036).

Additional Table 5 details the incidence of maternal and neonatal complications by type of labour. Maternal complications, as defined in Additional Table 2, were more frequent in IOL groups (36.2% and 39.3% in IOL group at 40 GW and 41 GW respectively vs 19.1% in SOL, *p* < 0.001). Postpartum haemorrhage (PPH) was the most frequent complication after CS and OVD (2.6% vs 5.7% vs 1.9% respectively, *p* = 0.001). The incidence of newborn complications was higher in births with IOL at 40 GW when compared to SOL (22.4% vs 13.4%, *p* < 0.001), particularly admissions to Special Care Baby Unit (15.8% in IOL at 40 GW vs 10.7% in IOL at 41 GW vs 8.6% in SOL group, *p* < 0.001). Newborn infections, neurological complications and respiratory distress syndrome were significantly more frequent in the group with IOL at 40 GW than SOL (respectively, 5.0% vs 2.2%, *p* = 0.002; 3.1% vs 1.3%, *p* = 0.009; 2.4% vs 0.9%, *p* = 0.002). Perinatal deaths and stillbirth rates were low across all groups (less than five perinatal deaths in each group of which only one was a stillbirth in IOL at 41 GW, classified as macerated stillbirth).

In multivariate analysis (Table 2) with SOL as reference and controlling for age, parity, education, BMI and neonatal weight, both IOL groups were positively associated with higher odds of any negative birth outcome (AOR = 2.21, 95%CI = 1.75–2.77, *p* < 0.001 for IOL at 40 GW and AOR = 1.91, 95%CI = 1.47–2.48, *p* < 0.001 for IOL at 41 GW), all maternal complications (AOR = 2.18, 95%CI = 1.71–2.77, *p* < 0.001 for IOL at 40 GW and AOR = 2.34, 95%CI = 1.78–3.07, *p* < 0.001 for IOL at 41 GW) and CS (AOR = 2.75, 95%CI = 2.07–3.65, *p* < 0.001 for IOL at 40 GW and AOR = 3.01, 95%CI = 2.21–4.12, *p* < 0.001 for IOL at 41 GW). IOL at 40 GW was associated with a higher number of neonatal complications (AOR = 1.63, 95%CI = 1.24–2.14, *p* < 0.001) and IOL at 41 GW was positively associated with other  maternal complications than CS or OVD (AOR = 1.83, 95%CI = 1.19–2.80, *p* = 0.006).

### Secondary and sensitivity analyses

IOL at 40 GW was positively associated with increased numbers of negative birth outcomes (AOR = 1.95, 95%CI = 1.56–2.44, *p* < 0.001), maternal complications (AOR = 1.82, 95%CI = 1.44–2.30, *p* < 0.001), CS (AOR = 2.09, 95%CI = 1.60–2.74, *p* < 0.001), and neonatal complications (AOR = 1.58, 95%CI = 1.21–2.06, *p* < 0.001) when compared with IOL at 41 GW and SOL combined (Additional Table 7). No other significant association was found (Additional Tables 6 and 7; Additional Fig. 2).

In the comparison IOL at 40 GW vs IOL at 41 GW, the former group was associated with higher OVD (AOR = 2.55, 95%CI = 1.18–5.52, *p* = 0.017) and less maternal complications other than CS or OVD (AOR = 0.49, 95%CI = 0.28–0.87, *p* = 0.015), with a lower frequency of PPH and maternal near miss (respectively, 2.6% vs 5.7%, *p* = 0.033; and 0.7% vs 4.1%, *p* = 0.001) (Additional Tables 8 and 9).

Results of sensitivity analysis, which included additional women with oligohydramnios, APH and impaired foetal wellbeing complications from 41 + 0 GW (Additional Table 10), did not differ from the primary analysis with both IOL groups positively associated with higher odds of any negative birth outcome (Additional Table 11).

## Discussion

### Main findings

In this study in Sri Lanka the practice of elective IOL at 40 GW or induction at 41 GW compared to SOL in a low-risk population was not associated with a reduction in complicated birth outcomes for the mother and/or the newborn. Both IOL groups were also associated with increased odds of CS compared to SOL.

### Interpretation

Our findings are partially in line with the most recent Cochrane systematic review, confirming that there is evidence of higher OVD rate in IOL at 40 GW vs IOL at 41 GW [7]. Discrepancies between our results for CS rates and other studies [7, 9, 12, 14, 28] could be accounted for by differences in setting, study design, and different definitions of comparison groups. Our study was set in Sri Lanka and included recent data from a maternity hospital registry, evaluating optimal timing of IOL in routine circumstances in a LMIC setting at predefined GW. Only 9 of 30 RCTs included in the Cochrane review were conducted in LMIC, while 13 (43%) studies were published from 1960s-1980s [18]. Furthermore, comparison groups in the Cochrane review are not directly comparable since timing of IOL differed among included trials as well as group definition, timing, and monitoring of expectant management.

Moreover, while RCT would be the most appropriate study design to address the question of optimal timing of IOL, this design has potential limitations due to difficulties in masking the intervention and high number of women declined participation (73% in the US study and 78% in the Swedish study [10, 13]). The availability of a prospective database capturing characteristics and outcomes of each delivery provides the opportunity to easily monitor indicators over time and compare practices and results in a real-world setting.

Overall, findings of this study highlight the need for caution in generalizing the results of RCT conducted in high income settings to different clinical settings and populations. More studies should be conducted to further explore the ideal timing of IOL in LMICs.

### Strengths and limitations

To our knowledge this is the first published study on the association between timing of IOL and maternal and newborn outcomes in low-risk pregnancies in Sri Lanka. It is also the first study from a setting with limited resources reporting on the use of a prospective individual-patient database to analyse practices and outcomes of IOL [23]. This study contributes to current international and local debate on the appropriateness of IOL near term. These study findings are extremely relevant locally both for clinicians, researchers and policy makers, as IOL at 40 GW is a common practice in Sri Lanka and has a significant economic impact on the health system and healthcare resources.

We acknowledge some limitations of this study. As an observational study, we could only assess associations between IOL and birth outcomes and not causation. Generalizability of study results may be limited by the characteristics of the local context and population in this single centre study. Larger sample sizes are required to detect significant differences in rare adverse events including stillbirth or maternal or perinatal death. Although gestational age was mostly determined by ultrasound examination, for 12% of the included women gestational age was estimated by menstrual dating.

Socio-cultural background and women’s empowerment may have affected both requests for induction and the type of care offered by physicians. Specifically, early induction (IOL at 40 GW) occurred more often in women with a high level of education. Unmarried women, still subjected to social stigma in Sri Lanka [29], were significantly more represented in the group undergoing IOL at 41 GW. Thus, numbers of CS and neonatal complications may have been influenced by socio-economic status. Other authors have described similar results, where unmarried women could have limited access to care [29] while higher social status or economic condition is related to an increasing medicalization of birth [30, 31]. However, in our study, since these imbalances among groups affect results in different directions, there may be limited risk of bias.

Though results were corrected for confounders, we cannot exclude heterogeneity among groups. Nulliparous women were more frequent in induced groups where the highest frequency of CS was recorded. Also, the combination of prostaglandin, oxytocin and artificial rupture of membranes was more frequent used in IOL at 41 GW. Since nulliparity, combination of induction techniques and induction itself are associated with an increased risk of CS [1, 9], it is impossible to say whether the higher frequency of negative outcomes, maternal complications and CS in IOL groups is related to the interventions or have suffered from selection bias.

Furthermore, induced women may have differed on characteristics not captured or not reported in the data collection form (such as unreported small for gestation foetuses, mild oligohydramnios, etc.). We were not able to explore specific practices related to IOL (such as safe use of uterotonics, appropriate maternal-foetal monitoring or CS indications), therefore we cannot exclude a difference among the groups for these variables. We had no information on the level of women’s participation in the decision process during labour care, nor specific choices, inclinations or skills of operators which may have had a substantial role in the differences observed [26, 32–34]. Notably, most of the evidence that we actually rely on may have some of these biases. Observational studies may not capture these aspects of care, while RCT, even though controlling these with randomization, may suffer from study effect.

Finally, another limitation related to the database is the absence of timing for risk factor onset. Hence it was impossible to differentiate between high-risk pregnancies (with risk factors before 40 + 0 GW) and low risk women at 40 + 0 GW who developed complications due to prolonged pregnancy (after 40 + 0 GW). A sensitivity analysis was performed to assess this limitation and results showed that it did not affect the overall findings.

## Conclusions

In our study, women with low risk pregnancy who underwent elective induction at 40 GW or induction at 41 GW in Colombo, Sri Lanka were associated with an increased risk of negative birth outcomes (CS, OVD or any complication) compared to women with spontaneous onset of labour. These findings should be used to improve monitoring and routine practices in Sri Lanka, as well as to better understand the optimal timing of IOL in other settings with low resources where IOL is a frequent practice.

## Supplementary Information

**Additional file 1.**

## Data Availability

Anonymized data that support the findings of this study are available at the department of obstetrics of Faculty of Medicine, University of Colombo. Ethics Review Committee of the Faculty of Medicine has granted permission for the authors to access and use the database. Restrictions apply to the availability of these data and so are not publicly available. Data are however available from the authors upon reasonable request and with permission of Ethics Review Committee of the Faculty of Medicine, University of Colombo.

## Abbreviations

AOR: Adjusted odds ratio
APH: Antepartum haemorrhage
BMI: Body mass index
CI: Confidence interval
CS: Caesarean section
GW: Gestational weeks
IOL: Induction of labour
IUGR: Intrauterine growth restriction at ultrasonography
LMIC: Low and middle-income countries
NICE: National Institute for health and Care Excellence
OT: Operating theatre
OVD: Operative vaginal delivery
PPH: Postpartum haemorrhage
RCT: Randomized clinical trial
SGA: Small for gestational age
SOL: Spontaneous onset of labour
STROBE: STrengthening the Reporting of OBservational studies in Epidemiology
UK: United Kingdom
US: United States
WHO: World Health Organization
