# Supplementary material for: Outcomes of induction versus spontaneous onset of labour at 40 and 41 GW: findings from a prospective database, Sri Lanka
Source: BMC Pregnancy Childbirth. 2022 Jun 27;22:518. doi: 10.1186/s12884-022-04800-1 (PMC9235207; doi:10.1186/s12884-022-04800-1)
Supplement: Supplementary file 1 — Additional file 1. [file 12884_2022_4800_MOESM1_ESM.pdf]

OUTCOMES OF INDUCTION VERSUS SPONTANEOUS ONSET OF LABOUR AT 40 AND 41 GW: FINDINGS FROM A PROSPECTIVE DATABASE, SRI LANKA

SUPPORTING INFORMATION

Table of Contents

Additional table 1. STROBE Statement—Checklist 2
Additional table 2. Negative outcomes: list of maternal and neonatal complications 4
Additional table 3. Methods of cervical ripening and induction of labour 5
Additional table 4. Detailed numbers of births with negative outcomes by gestational age and onset of labour 6
Additional figure 1. Trend of cesarean section and vaginal operative delivery 7
Additional table 5. Incidence of maternal and neonatal complications1 by gestational age and onset of labour 8
Additional table 6. Comparison bewteen IOL at 40 GW vs IOL at 41 GW and SOL: characteristics and outcomes by gestational age and onset of labour 9
Additional figure 2. Comparison bewteen IOL at 40 GW vs IOL at 41 GW and SOL: Births with negative outcomes 11
Additional table 7. Comparison bewteen IOL at 40 GW vs IOL at 41 GW and SOL: Adjusted odds ratios for negative outcomes 12
Additional table 8. Comparison etween IOL at 40 GW vs IOL at 41 GW: characteristics and outcomes by gestational age and onset of labour 13
Additional table 9. Comparison etween IOL at 40 GW vs IOL at 41 GW: Adjusted odds ratios for negative outcomes 16
Additional table 10. Description of additional women included in sensitivity analysis[1] only 17
Additional table 11. Sensitivity analysis: adjusted odds ratios for negative outcomes 18

Additional table 1. STROBE Statement—Checklist

STROBE Statement—Checklist of items that should be included in reports of ***cross-sectional studies***

	Item No	Recommendation	Page
**Title and abstract**	1	(*a*) Indicate the study's design with a commonly used term in the title or the abstract	3
		(*b*) Provide in the abstract an informative and balanced summary of what was done and what was found	3
**Introduction**			
Background/rationale	2	Explain the scientific background and rationale for the investigation being reported	4-5
Objectives	3	State specific objectives, including any prespecified hypotheses	5
**Methods**			
Study design	4	Present key elements of study design early in the paper	6
Setting	5	Describe the setting, locations, and relevant dates, including periods of recruitment, exposure, follow-up, and data collection	6
Participants	6	(*a*) Give the eligibility criteria, and the sources and methods of selection of participants	6-7
Variables	7	Clearly define all outcomes, exposures, predictors, potential confounders, and effect modifiers. Give diagnostic criteria, if applicable	7
Data sources/ measurement	8*	For each variable of interest, give sources of data and details of methods of assessment (measurement). Describe comparability of assessment methods if there is more than one group	6
Bias	9	Describe any efforts to address potential sources of bias	7
Study size	10	Explain how the study size was arrived at	6
Quantitative variables	11	Explain how quantitative variables were handled in the analyses. If applicable, describe which groupings were chosen and why	-
Statistical methods	12	(*a*) Describe all statistical methods, including those used to control for confounding	7
		(*b*) Describe any methods used to examine subgroups and interactions	7
		(*c*) Explain how missing data were addressed	6-7
		(*d*) If applicable, describe analytical methods taking account of sampling strategy	7
		(*e*) Describe any sensitivity analyses	7
**Results**			
Participants	13*	(a) Report numbers of individuals at each stage of study—eg numbers potentially eligible, examined for eligibility, confirmed eligible, included in the study, completing follow-up, and analysed	8
		(b) Give reasons for non-participation at each stage	-
		(c) Consider use of a flow diagram	8
Descriptive data	14*	(a) Give characteristics of study participants (eg demographic, clinical, social) and information on exposures and potential confounders	9
		(b) Indicate number of participants with missing data for each variable of interest	9
Outcome data	15*	Report numbers of outcome events or summary measures	10-11
Main results	16	(*a*) Give unadjusted estimates and, if applicable, confounder-adjusted estimates and their precision (eg, 95% confidence interval). Make clear which confounders were adjusted for and why they were included	11-12
		(*b*) Report category boundaries when continuous variables were categorized	9-12
		(*c*) If relevant, consider translating estimates of relative risk into absolute risk for a meaningful time period	-
Other analyses	17	Report other analyses done—eg analyses of subgroups and interactions, and sensitivity analyses	12
**Discussion**			
Key results	18	Summarise key results with reference to study objectives	13

Limitations	19	Discuss limitations of the study, taking into account sources of potential bias or imprecision. Discuss both direction and magnitude of any potential bias	13-14
Interpretation	20	Give a cautious overall interpretation of results considering objectives, limitations, multiplicity of analyses, results from similar studies, and other relevant evidence	14-15
Generalisability	21	Discuss the generalisability (external validity) of the study results	14-15
**Other information**			
Funding	22	Give the source of funding and the role of the funders for the present study and, if applicable, for the original study on which the present article is based	15

*Give information separately for exposed and unexposed groups.

Note: An Explanation and Elaboration article discusses each checklist item and gives methodological background and published examples of transparent reporting. The STROBE checklist is best used in conjunction with this article (freely available on the Web sites of PLoS Medicine at http://www.plosmedicine.org/, Annals of Internal Medicine at http://www.annals.org/, and Epidemiology at http://www.epidem.com/). Information on the STROBE Initiative is available at www.strobe-statement.org.

Additional table 2. Negative outcomes: list of maternal and neonatal complications

Negative outcomes	
**Maternal complications**	**Neonatal complications**
Abruptio placentae<br>Amniotic fluid embolisms<br>Caesarean section<br>Cord prolapse<br>Hysterectomy<br>Intensive Care Unit admission<br>Maternal death<br>Near miss [1]<br>Operative theatre (OT) admission after delivery<br>Operative vaginal delivery<br>Other complications not further specified<br>Perineal tears 3rd-4th degree<br>Postpartum haemorrhage [2]<br>Sepsis or severe infection<br>Uterine rupture	Apgar ≤5 at 10'<br>Asphyxia [3]<br>Jaundice with exchange transfusion<br>Major birth trauma [4]<br>Meconium aspiration syndrome<br>Need of feeding support<br>Neonatal intensive care unit admission<br>Neonatal length of stay ≥ 10 days<br>Other complications not further specified<br>Other infection, fever (no sepsis)<br>Other respiratory conditions [5]<br>Perinatal deaths included stillbirth [6]<br>Phototherapy > 24h [7]<br>Respiratory distress syndrome [8]<br>Seizures, ventricular hemorrhage and other major neurological complications [9]<br>Semi-intensive care baby unit admission<br>Sepsis<br>Ventilation in delivery room

Notes: [1] Near miss: severe disease (severe postpartum haemorrhage, pre-eclampsia, eclampsia, sepsis, uterine rupture, severe complications of abortion), or critical interventions (Intensive Care Unit admission, intervention radiology, laparotomy, blood transfusion) or organ dysfunction. [2] Postpartum haemorrhage (PPH): minor PPH (blood loss of 500-999 ml) or severe PPH (blood loss ≥ 1000 ml or any bleeding with hypotension or tachycardia or blood transfusion) or massive PPH (lost of ≥40% of blood volume calculated as body weight(kg)/12). [3] Asphyxia: no spontaneous start of breathing, ventilation for at least 30 sec and/or thoracic compressions as in international guidelines or any drug. [4] Major birth trauma: brachial plexus injury/arm palsy, fractures at any site, sub-aponeurotic (subgaleal) hemorrhage. [5] Other respiratory conditions: any respiratory condition other than respiratory complications listed in the table. [6] Stillbirth: both macerated and fresh stillbirth based on clinical evaluation. [7] Phototherapy > 24h: included as a proxy of other neonatal complications such as large for gestational age. [8] Respiratory distress syndrome: respiratory distress lasting more than 24 hours. [9] Neurological complications: seizures, ventricular hemorrhage or other severe neurological complications.

Additional table 3. Methods of cervical ripening and induction of labour

	IOL at 40 GW (40+0 to 40+6) N=456		IOL at 41 GW (41+0 to 41+6) N=318		
	**n**	**%**	**n**	**%**	**p-value**
Prostaglandin	222	48.79	137	43.63	0.124
Artificial rupture of membranes	59	12.97	32	10.19	0.222
Foley and artificial rupture of membranes or oxytocin	35	7.69	36	11.46	0.084
Prostaglandin, oxytocin and artificial rupture of membranes	31	6.81	55	17.52	**<0.001**
Foley	38	8.35	20	6.37	0.288
Artificial rupture of membranes and oxytocin	29	6.37	14	4.46	0.242
Prostaglandin and artificial rupture of membranes	12	2.64	7	2.23	0.703
Oxytocin	18	3.96	6	1.91	0.104
Foley and prostaglandin	9	1.98	6	1.91	0.931
Other	2	0.44	1	0.32	>0.99

Abbreviations: IOL= induction of labour.

Additional table 4. Detailed numbers of births with negative outcomes by gestational age and onset of labour

	IOL at 40 GW (40+0 to 40+6) N=456 n (%)	IOL at 41 GW (41+0 to 41+6) N=318 n (%)	SOL (40+0 to 41+6) N=1,585 n (%)
**Births with negative outcomes**	**215 (47.1)** [1]	**144 (45.2)** [2]	**428 (27.1)**
**Caesarean section (CS)**	**116 (25.4)** [1]	**91 (28.6)** [2]	**163 (10.3)**
CS and other complications	37/116 (32.0)	34/91 (37.4)	66 /163 (40.5)
**Operative vaginal delivery (OVD)**	**32 (7.0)**	**9 (2.8)**	**77 (4.9)**
OVD and other complications	14/32 (43.8)	4/9 (44.5)	28/77 (36.4)
**Any other complication** [3]	**67 (14.7)**	**44 (13.8)**	**188 (11.9)**
Other maternal complications [3]	17/67 (25.4)	25/44 (56.8) [2]	63/188 (33.5)
Other neonatal complications [3]	54/67 (80.6) [1]	25/44 (56.8)	136/188 (72.3)

Notes: [1] Significant p value ($p<0.05$) in the comparison IOL at 40 GW vs SOL. [2] Significant p value ($p<0.05$) in the comparison IOL at 41 GW vs SOL. [3] Maternal and neonatal complications are defined in additional table 2.
Abbreviations: GW= gestational weeks; IOL= induction of labour; SOL= spontaneous onset of labour.

Additional figure 1. Trend of cesarean section and vaginal operative delivery

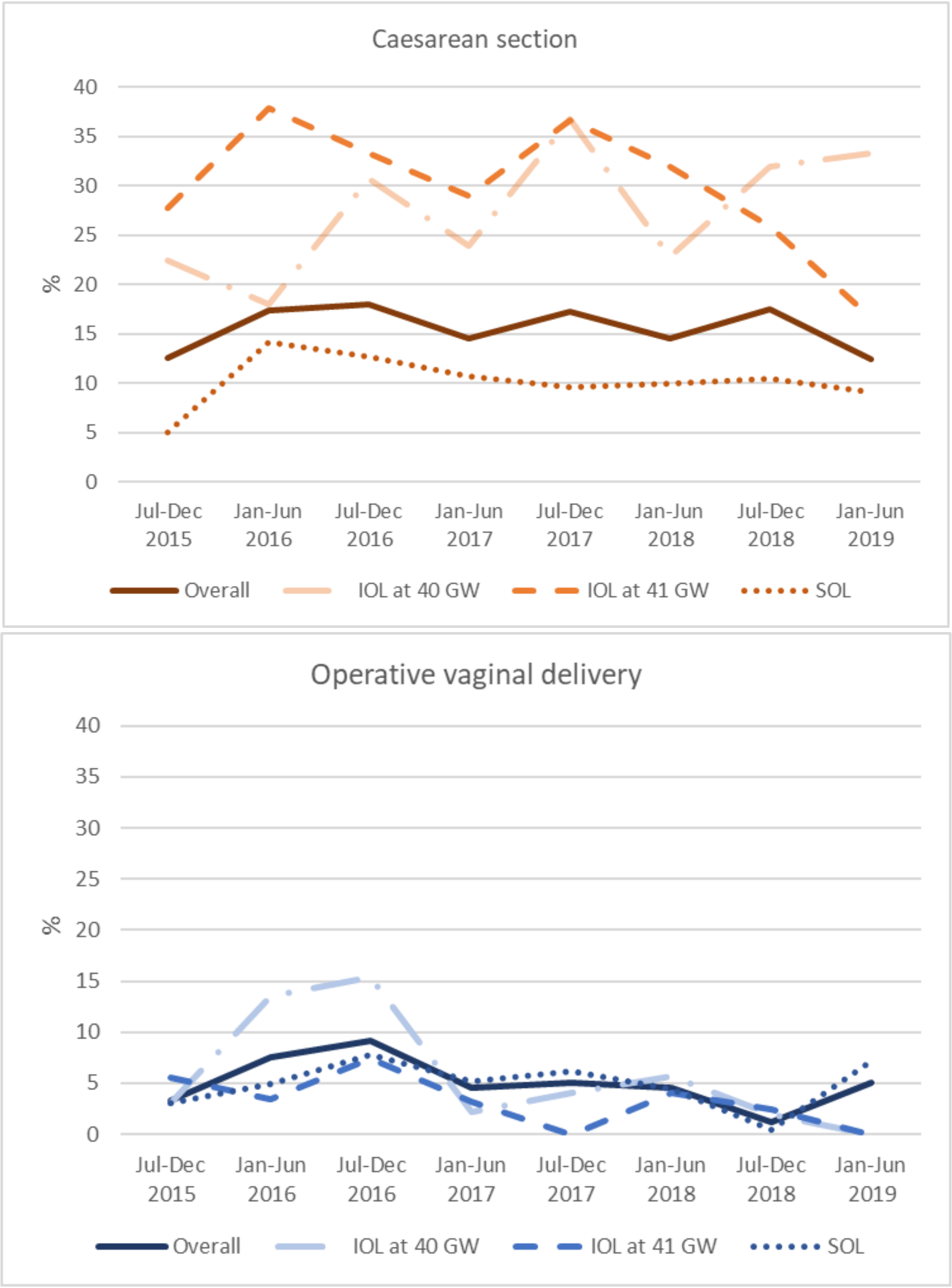

Abbreviations: CS= caesarean section; GW= gestational weeks; IOL= induction of labour; SOL= spontaneous onset of labour.

Additional files

Additional table 5. Incidence of maternal and neonatal complications1 by gestational age and onset of labour

	IOL at 40 GW (40+0 to 40+6) N=456 n (%)	IOL at 41 GW (41+0 to 41+6) N=318 n (%)	SOL (40+0 to 41+6) N=1,585 n (%)
**All maternal complications [1]**	**165 (36.2) [11]**	**125 (39.3) [12]**	**303 (19.1)**
CS	116 (25.4) [11]	91 (28.6) [12]	163 (10.3)
Operative vaginal delivery	32 (7.0)	9 (2.8)	77 (4.9)
**Other maternal complications (any of the below)**	**23 (5.0)**	**33 (10.4) [12]**	**87 (5.5)**
Maternal death	0	0	0
PPH [2]	12 (2.6)	18 (5.7) [12]	30 (1.9)
Minor PPH [2]	10 (2.2)	13 (4.1) [12]	16 (1.0)
Severe or massive PPH [2]	2 (0.4)	5 (1.6)	14 (0.9)
Near miss [3]	3 (0.7)	13 (4.1) [12]	22 (1.4)
Perineal tears 3rd-4th degree	5 (1.1)	8 (2.5)	38 (2.4)
Sepsis or severe infection	1 (0.2)	4 (1.3)	12 (0.8)
ICU admission	1 (0.2)	1 (0.3)	7 (0.4)
OT admission after delivery	0	0	2 (0.1)
Abruptio placentae	0	1 (0.3)	0
Other complications not further specified	5 (1.1)	4 (1.3)	6 (0.4)
**All neonatal complications [1]**	**102 (22.4) [11]**	**56 (17.6)**	**213 (13.4)**
Stillbirth [4]	0	1 (0.3)	0
Perinatal deaths included stillbirth [4]	1 (0.2)	2 (0.6)	5 (0.5)
SCBU admission	72 (15.8) [11]	34 (10.7)	136 (8.6)
Need of feeding support	30 (6.6) [11]	25 (7.9) [12]	62 (3.9)
Other infection, fever (no sepsis)	23 (5.0) [11]	9 (2.8)	35 (2.2)
Neurological complications [5]	14 (3.1) [11]	3 (0.9)	20 (1.3)
Respiratory distress syndrome [6]	11 (2.4) [11]	4 (1.3)	15 (0.9)
Ventilation in delivery room	11 (2.4)	4 (1.3)	24 (1.5)
Apgar ≤5 at 10’	10 (2.2)	4 (1.3)	23 (1.5)
Meconium aspiration syndrome	8 (1.8)	1 (0.3)	20 (1.3)
Neonatal length of stay ≥ 10 days	5 (1.1)	3 (0.9)	5 (0.3)
NICU admission	1 (0.2)	1 (0.3)	4 (0.3)
Asphyxia [7]	5 (1.1)	0	9 (0.6)
Other respiratory conditions [8]	4 (0.9)	0	6 (0.4)
Phototherapy > 24h [9]	3 (0.7)	0	6 (0.4)
Jaundice with exchange transfusion	2 (0.4)	0	4 (0.3)
Major birth trauma [10]	0	0	1 (0.1)
Sepsis	0	0	1 (0.1)
Other complications not further specified	6 (1.3)	4 (1.3)	8 (0.5)

Notes: [1] Maternal and neonatal complications are defined in additional table 2. No woman reported cord prolapse, hysterectomy or uterine rupture thus these complications were not reported in the table. [2] Postpartum haemorrhage (PPH): minor PPH (blood loss of 500-999 ml) or severe PPH (blood loss ≥ 1000 ml or any bleeding with hypotension or tachycardia or blood transfusion) or massive PPH (lost of ≥40% of blood volume calculated as body weight(kg)/12). [3] Near miss: severe disease (severe postpartum haemorrhage, severe pre-eclampsia, eclampsia, sepsis, uterine rupture, severe complications of abortion), or critical interventions (Intensive Care Unit admission, intervention radiology, laparotomy, blood transfusion) or organ dysfunction. [4] Stillbirth: both macerated and fresh stillbirth based on clinical evaluation; the only case of stillbirth that occurred in the study was a macerated stillbirth. [5] Neurological complications: seizures, ventricular hemorrhage or other severe neurological complications. [6] Respiratory distress syndrome: respiratory distress lasting more than 24 hours. [7] Asphyxia: no spontaneous start of breathing, ventilation for at least 30 sec and/or thoracic compressions as in international guidelines or any drug. [8] Other respiratory conditions: any respiratory condition other than respiratory complications listed in the table. [9] Phototherapy > 24h: included as a proxy of other neonatal complications such as large for gestational age. [10] Major birth trauma: brachial plexus injury/arm palsy, fractures at any site, sub-aponeurotic (subgaleal) hemorrhage. [11] Significant p value ($p<0.05$) in the comparison IOL at 40 GW vs SOL. [12] Significant p value ($p<0.05$) in the comparison IOL at 41 GW vs SOL.

Abbreviations: CS= caesarean section; GW= gestational weeks; ICU= Intensive Care Unit; IOL= induction of labour; NICU=Neonatal intensive care unit; OT= operative theatre; PPH= postpartum haemorrhage; SCBU= Special Care Baby Unit; SOL= spontaneous onset of labour.

Additional table 6. Comparison bewteen IOL at 40 GW vs IOL at 41 GW and SOL: characteristics and outcomes by gestational age and onset of labour

	IOL at 40 GW<br>N=456<br>n (%)	IOL at 41 GW and SOL<br>N=1,903<br>n (%)
**Socio-demographic and clinical characteristics**		
Maternal Age		
< 35 years	401 (87.9)	1,714 (90.1)
≥ 35 years	55 (12.1)	189 (9.9)
Education		
None	1 (0.2)	4 (0.2)
Primary	10 (2.2)	30 (1.6)
Secondary	353 (77.4) [1]	1,607 (84.4)
Higher	91 (20.0) [1]	257 (13.5)
Missing	1 (0.2)	5 (0.3)
Working status		
Working	81 (17.8)	275 (14.5)
Housewife	370 (81.2)	1,614 (84.8)
Missing	5 (1.1)	14 (0.7)
Marital status		
Married	451 (98.9)	1,875 (98.5)
Unmarried	3 (0.6)	21 (1.1)
Unmarried living together	1 (0.2)	2 (0.1)
Missing	1 (0.2)	5 (0.3)
Parity		
0	260 (57.0) [1]	952 (50.0)
≥1	196 (43.0) [1]	951 (50.0)
Asian criteria-based BMI [2]		
Underweight (<18.4)	38 (8.2)	192 (10.1)
Normal (18.5-22.9)	312 (68.4)	1,251 (65.7)
Overweight (23-27.4)	106 (23.2)	460 (24.2)
Operator delivering care		
Nurse	200 (43.9) [1]	1,015 (53.3)
Midwife	110 (24.1)	532 (28.0)
House Officer	4 (0.9)	28 (1.5)
Mid-level staff [3]	140 (30.7) [1]	320 (16.8)
Consultant	2 (0.4)	4 (0.2)
Missing	0	4 (0.2)
Weight at birth		
<2,000	0	0
2,000 to 2,499	13 (2.8)	58 (3.0)
2,500 to 3,499	374 (82.0)	1,524 (80.1)
3,500 to 4,000	57 (12.5)	295 (15.5)
>4,000	11 (2.4) [1]	21 (1.1)
Missing	1 (0.2)	5 (0.3)
**Outcomes**		
**Births with negative outcomes (any of the below)**	215 (47.1) [1]	572 (30.1)
Caesarean section	116 (25.4) [1]	254 (13.3)
Operative vaginal delivery	32 (7.0) [1]	86 (4.5)
**Maternal complications [4]**	23 (5.0)	120 (6.3)
Maternal death	0	0
PPH [5]	12 (2.6)	48 (2.5)
Minor PPH [5]	10 (2.2)	29 (1.5)
Severe or massive PPH [5]	2 (0.4)	19 (1.0)
Near miss [6]	3 (0.7)	35 (1.8)

Perineal tears 3rd-4th degree	5 (1.1)	46 (2.41)
Sepsis or severe infection	1 (0.2)	16 (0.8)
ICU admission	1 (0.2)	8 (0.4)
OT after delivery	0	2 (0.1)
Abruptio placentae	0	1 (0.1)
Other complications not further specified	5 (1.1)	10 (0.5)
**Neonatal complications [4]**	102 (22.4) [1]	269 (14.1)
Stillbirth [7]	0	1 (0.1)
Perinatal deaths included stillbirth [7]	1 (0.2)	4 (0.2)
SCBU admission	72 (15.8) [1]	170 (8.9)
Need of feeding support	30 (6.6)	87 (4.6)
Other infection, fever (no sepsis)	23 (5.0) [1]	44 (2.3)
Neurological complications [8]	14 (3.1) [1]	23 (1.2)
Respiratory distress syndrome [9]	11 (2.4) [1]	19 (1.0)
Ventilation in delivery room	11 (2.4)	28 (1.5)
Apgar ≤5 at 10'	10 (2.2)	27 (1.4)
Meconium aspiration syndrome	8 (1.8)	21 (1.1)
Neonatal length of stay ≥ 10 days	5 (1.1)	8 (0.4)
Asphyxia [10]	5 (1.1)	9 (0.5)
NICU admission	1 (0.2)	5 (0.3)
Phototherapy > 24h [11]	3 (0.7)	6 (0.3)
Jaundice with exchange transfusion	2 (0.4)	4 (0.2)
Other respiratory conditions [12]	4 (0.9)	6 (0.3)
Major birth trauma [13]	0	1 (0.1)
Sepsis	0	1 (0.1)
Other complications not further specified	6 (1.3)	12 (0.6)

Notes: [1] Significant p value ($p<0.05$) in the comparison IOL at 40 GW vs IOL at 41 GW and SOL; [2] Asian criteria-based BMI as defined by the WHO Expert Consultation in the document "Appropriate body-mass index for Asian populations and its implications for policy and intervention strategies". *Lancet*. 2004 Jan 10;363(9403):157-63. [3] Mid-level staff defined as Senior House Officer or Registrar. [4] Maternal and neonatal complications are defined in additional table 2. No woman reported cord prolapse, hysterectomy or uterine rupture thus these complications were not reported in the table. [5] Postpartum haemorrhage (PPH): minor PPH (blood loss of 500-999 ml) or severe PPH (blood loss ≥ 1000 ml or any bleeding with hypotension or tachycardia or blood transfusion) or massive PPH (lost of ≥40% of blood volume calculated as body weight(kg)/12). [6] Near miss: severe disease (severe postpartum haemorrhage, severe pre-eclampsia, eclampsia, sepsis, uterine rupture, severe complications of abortion), or critical interventions (Intensive Care Unit admission, intervention radiology, laparotomy, blood transfusion) or organ dysfunction. [7] Stillbirth: both macerated and fresh stillbirth based on clinical evaluation; the only case of stillbirth that occurred in the study was a macerated stillbirth. [8] Neurological complications: seizures, ventricular hemorrhage or other severe neurological complications. [9] Respiratory distress syndrome: respiratory distress lasting more than 24 hours. [10] Asphyxia: no spontaneous start of breathing, ventilation for at least 30 sec and/or thoracic compressions as in international guidelines or any drug. [11] Phototherapy > 24h: included as a proxy of other neonatal complications such as large for gestational age. [12] Other respiratory conditions: any respiratory condition other than respiratory complications listed in the table. [13] Major birth trauma: brachial plexus injury/arm palsy, fractures at any site, sub-aponeurotic (subgaleal) hemorrhage.

Abbreviation: BMI= body mass index; GW= gestational weeks; ICU: Intensive Care Unit; IOL= Induction of labour; NICU=Neonatal intensive care unit; OT= operative theatre; PPH= Postpartum haemorrhage; SCBU= Special Care Baby Unit; SOL= Spontaneous onset of labour.

Additional figure 2. Comparison bewteen IOL at 40 GW vs IOL at 41 GW and SOL: Births with negative outcomes

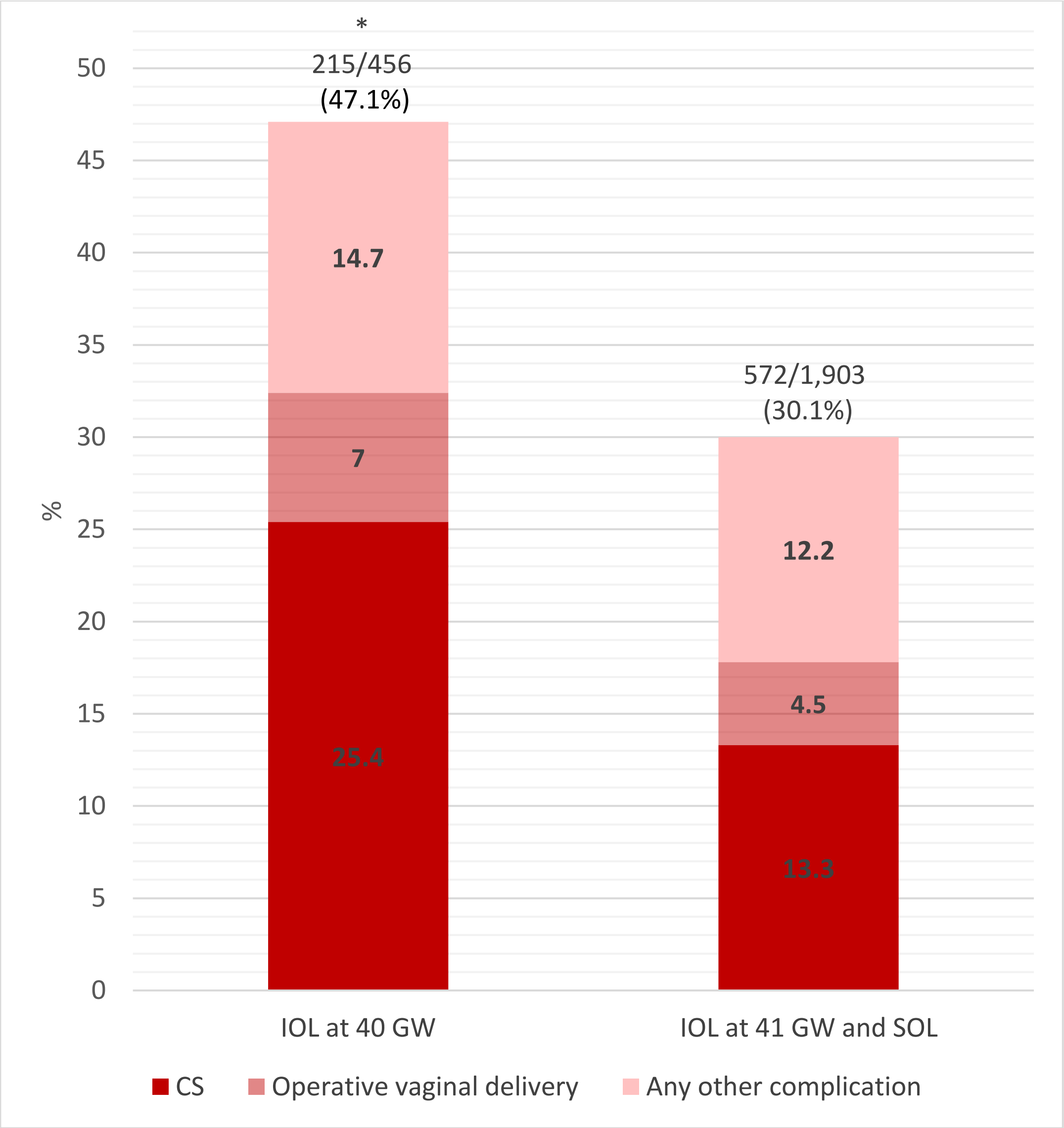

Notes: * Significant p value (p<0.05) in the comparison between the two groups.
Abbreviations: CS = Cesarean section; GW= Gestational weeks; IOL= Induction of labour; SOL= Spontaneous onset of labour.

Additional table 7. Comparison bewteen IOL at 40 GW vs IOL at 41 GW and SOL: Adjusted odds ratios for negative outcomes

	**IOL at 40 GW N=456 n (%)**		**SOL and IOL at 41 GW N=1,903 n (%)**
	**OR (95% CI)**	**p value**	
Any negative outcome	1.95 (1.56-2.44)	**<0.001**	Ref
All maternal complications	1.82 (1.44-2.30)	**<0.001**	Ref
CS	2.09 (1.60-2.74)	**<0.001**	Ref
Operative vaginal delivery	1.42 (0.93-2.19)	0.111	Ref
Other maternal complications	0.77 (0.48-1.22)	0.265	Ref
All neonatal complications	1.58 (1.21-2.06)	**<0.001**	Ref

Notes: ORs are adjusted for age, parity, education, BMI and neonatal weight.

Abbreviations: CS= caesarean section; GW= gestational week; IOL= induction of labour; SOL= spontaneous onset of labour.

Additional table 8. Comparison etween IOL at 40 GW vs IOL at 41 GW: characteristics and outcomes by gestational age and onset of labour

Population	IOL at 40 GW (40+0 to 40+6) N=456 n (%)	IOL at 41 GW (41+0 to 41+6) N=318 n (%)
**Socio-demographic and clinical characteristics**		
Maternal Age		
< 35 years	401 (87.9)	290 (91.2)
≥ 35 years	55 (12.1)	28 (8.8)
Education		
None	1 (0.2)	2 (0.6)
Primary	10 (2.2)	4 (1.3)
Secondary	353 (77.4)	266 (83.6) [1]
Higher	91 (20.0)	46 (14.5)
Missing	1 (0.2)	0
Working status		
Working	81 (17.8)	48 (15.1)
Housewife	370 (81.1)	270 (84.9)
Missing	5 (1.1)	0
Marital status		
Married	451 (98.9)	311 (97.8)
Unmarried	3 (0.7)	7 (2.2)
Unmarried living together	1 (0.2)	0
Missing	1 (0.2)	0
Parity		
0	260 (57.0)	198 (62.3)
≥1	196 (43.0)	120 (37.7)
Asian criteria-based BMI [2]		
Underweight (<18.4)	38 (8.3)	33 (10.4)
Normal (18.5-22.9)	312 (68.4)	190 (59.7) [1]
Overweight (23-27.4)	106 (23.2)	95 (29.9) [1]
Operator delivering care		
Nurse	200 (43.9)	116 (36.5) [1]
Midwife	110 (24.1)	101 (31.8) [1]
House Officer	4 (0.9)	4 (1.3)
Mid-level staff [3]	140 (30.7)	96 (30.2)
Consultant	2 (0.4)	1 (0.3)
Missing	0	0
Neonatal weight at birth		
<2000	0	0
2000 to 2499	13 (2.9)	3 (0.9)
2500 to 3499	374 (82.0)	246 (77.4)
3500 to 4000	57 (12.5)	61 (19.2) [1]
>4000	11 (2.4)	8 (2.5)
Missing	1 (0.2)	0

Outcomes		
**Births with negative outcomes (any of the below)**	**215 (47.1)**	**144 (45.2)**
Caesarean section	116 (25.4)	91 (28.6)
Operative vaginal delivery	32 (7.0)	9 (2.8) [1]
**Maternal complications [4]**	**23 (5.0)**	**33 (10.4) [1]**
Maternal death	0	0
PPH [5]	12 (2.6)	18 (5.7) [1]
Minor PPH [5]	10 (2.2)	13 (4.1)
Severe or massive PPH [5]	2 (0.4)	5 (1.6)
Near miss [6]	3 (0.7)	13 (4.1) [1]
Perineal tears 3rd-4th degree	5 (1.1)	8 (2.5)
Sepsis or severe infection	1 (0.2)	4 (1.3)
ICU admission	1 (0.2)	1 (0.3)
OT admission after delivery	0	0
Abruptio placentae	0	1 (0.3)
Other complications not further specified	5 (1.1)	4 (1.3)
**Neonatal complications [4]**	**102 (22.4)**	**56 (17.6)**
Stillbirth [7]	0	1 (0.3)
Perinatal deaths included stillbirth [7]	1 (0.2)	2 (0.6)
SCBU admission	72 (15.8)	34 (10.7) [1]
Need of feeding support	30 (6.6)	25 (7.9)
Other infection, fever (no sepsis)	23 (5.0)	9 (2.8)
Neurological complications [8]	14 (3.1)	3 (0.9)
Respiratory distress syndrome [9]	11 (2.4)	4 (1.3)
Ventilation in delivery room	11 (2.4)	4 (1.3)
Apgar ≤5 at 10’	10 (2.2)	4 (1.3)
Meconium aspiration syndrome	8 (1.8)	1 (0.3)
Neonatal length of stay ≥ 10 days	5 (1.1)	3 (0.9)
NICU admission	1 (0.2)	1 (0.3)
Asphyxia [10]	5 (1.1)	0
Other respiratory conditions [11]	4 (0.9)	0
Phototherapy > 24h [12]	3 (0.7)	0
Jaundice with exchange transfusion	2 (0.4)	0
Major birth trauma [13]	0	0
Sepsis	0	0
Other complications not further specified	6 (1.3)	4 (1.3)

Notes: [1] Significant p value (p<0.05) in the comparison IOL at 40 GW IOL at 41 GW. [2] Asian criteria-based BMI as defined by the WHO Expert Consultation in the document “Appropriate body-mass index for Asian populations and its implications for policy and intervention strategies”. *Lancet*. 2004 Jan 10;363(9403):157-63. [3] Mid-level staff defined as Senior House Officer or Registrar. [4] Maternal and neonatal complications are defined in additional table 2. No woman reported cord prolapse, hysterectomy or uterine rupture thus these complications were not reported in the table. [5] Postpartum haemorrhage (PPH): minor PPH (blood loss of 500-999 ml) or severe PPH (blood loss ≥ 1000 ml or any bleeding with hypotension or tachycardia or blood transfusion) or massive PPH (lost of ≥40% of blood volume calculated as body weight(kg)/12). [6] Near miss: severe disease (severe postpartum haemorrhage, severe pre-eclampsia, eclampsia, sepsis, uterine rupture, severe complications of abortion), or critical interventions (Intensive Care Unit admission, intervention radiology, laparotomy, blood transfusion) or organ dysfunction. [7] Stillbirth: both macerated and fresh stillbirth based on clinical evaluation; the only case of stillbirth that occurred in the study was a macerated stillbirth. [8] Neurological complications: seizures, ventricular hemorrhage or other severe neurological complications. [9] Respiratory distress syndrome: respiratory distress lasting more than 24 hours. [10] Asphyxia: no spontaneous start of breathing, ventilation for at least 30 sec and/or thoracic compressions as in international guidelines or any drug. [11] Other respiratory conditions: any respiratory condition other than respiratory complications listed in the table. [12] Phototherapy > 24h: included as a proxy of other neonatal complications such as large for gestational age. [13] Major birth trauma: brachial plexus injury/arm palsy, fractures at any site, sub-aponeurotic (subgaleal) hemorrhage.

Abbreviations: BMI= body mass index; GW= gestational weeks; ICU= Intensive Care Unit; IOL= induction of labour; NICU=Neonatal intensive care unit; OT= operative theatre; PPH= postpartum haemorrhage; SCBU= Special Care Baby Unit; SOL= spontaneous onset of labour.

Additional files

Additional table 9. Comparison etween IOL at 40 GW vs IOL at 41 GW: Adjusted odds ratios for negative outcomes

	**IOL at 40 GW N=456 n (%)**		**IOL at 41 GW N=318 n (%)**
		**p value**	
Any negative outcome	1.17 (0.86-1.60)	0.325	Ref
All maternal complications	0.94 (0.68-1.29)	0.703	Ref
Caesarean section	0.91 (0.64-1.29)	0.602	Ref
Operative vaginal delivery	2.55 (1.18-5.52)	**0.017**	Ref
Other maternal complications	0.49 (0.28-0.87)	**0.015**	Ref
All neonatal complications	1.34 (0.92-1.96)	0.129	Ref

Notes: Ors are adjusted for age, parity, education, BMI and neonatal weight.

Abbreviations: GW= gestational week; IOL= induction of labour; SOL= spontaneous onset of labour.

Additional table 10. Description of additional women included in sensitivity analysis[1] only

	IOL at 41 GW<br>N=3<br>n (%)	SOL<br>N=1<br>n (%)
**Socio-demographic and clinical characteristics**		
Age <35 years	3 (100%)	1 (100%)
Education		
Secondary	2 (66.7%)	0
Higher	1 (33.3%)	1 (100%)
Working status		
Working	0	1 (100%)
Housewife	3 (100%)	0
Parity		
0	2 (66.7%)	1 (100%)
≥1	1 (33.3%)	0
Asian criteria-based BMI [2]		
Normal (18.5-22.9)	1 (33.3%)	1 (100%)
Overweight (23-27.4)	2 (66.7%)	0
Neonatal weight at birth (grams)		
2500 to 3499	2 (66.7%)	0
3500 to 4000	1 (33.3%)	1 (100%)
**Outcomes**		
**Any negative outcomes (any of the below)**	**3 (100%)**	**1 (100%)**
**Maternal complications**	**3 (100%)**	**1 (100%)**
CS	2 (66.7%)	1 (100%)
Oligohydramnios	1 (33.3%)	0
APH	0	1 (100%)
Abruptio placentae	1 (33.3%)	0
**Neonatal complications**	**3 (100%)**	**0**
Signs of potentially impaired foetal well being [1]	2 (66.7%)	0
Ventilaton in the delivery room	1 (33.3%)	0

Notes: [1] Women from 41+0 GW with hypertensive disorders, chorioamnionitis, oligohydramnios, APH, signs of potentially impaired foetal well being (non-reassuring or pathological cardiotocography, reduced foetal movement, meconium stained amniotic fluid) were included in sensitivity analysis. Only complications with at least one case are shown in the table. [2] Asian criteria-based BMI as defined by the WHO Expert Consultation in the document “Appropriate body-mass index for Asian populations and its implications for policy and intervention strategies”. *Lancet*. 2004 Jan 10;363(9403):157-63.

Abbreviations: APH= antepartum haemorrhage; BMI= body mass index; CS= caesarean section; GW= gestational week; IOL= induction of labour; SOL= spontaneous onset of labour.

Additional table 11. Sensitivity analysis: adjusted odds ratios for negative outcomes

	IOL at 40 GW (40+0 to 40+6) N=456		IOL at 41 GW (41+0 to 41+6) N=321		SOL (40+0 to 41+6) N=1,586
	Adj OR (95% CI)	p value	Adj OR (95% CI)	p value	
Any negative outcome	2.20 (1.75-2.77)	**<0.001**	1.94 (1.50-2.52)	**<0.001**	Ref
All maternal complications	2.17 (1.71-2.77)	**<0.001**	2.39 (1.82-3.13)	**<0.001**	Ref
Caesarean section	2.73 (2.06-3.63)	**<0.001**	3.06 (2.24-4.16)	**<0.001**	Ref
Operative vaginal delivery	1.27 (0.82-1.98)	0.281	0.47 (0.23-1.96)	**0.039**	Ref
Other maternal complications	0.87 (0.54-1.40)	0.567	1.84 (1.20-2.81)	**0.005**	Ref
All neonatal complications	1.63 (1.24-2.14)	**<0.001**	1.23 (0.88-1.71)	0.213	Ref

Notes: ORs are adjusted for age, parity, education, BMI and neonatal weight.

Abbreviations: GW= gestational weeks; IOL= induction of labour; SOL= spontaneous onset of labour.
